# Targeting oxytocin receptor (Oxtr)-expressing neurons in the lateral septum to restore social novelty in autism spectrum disorder mouse models

**DOI:** 10.1038/s41598-020-79109-0

**Published:** 2020-12-17

**Authors:** Machi Horiai, Ayano Otsuka, Shizu Hidema, Yuichi Hiraoka, Ryotaro Hayashi, Shinji Miyazaki, Tamio Furuse, Hiroaki Mizukami, Ryoichi Teruyama, Masaru Tamura, Haruhiko Bito, Yuko Maejima, Kenju Shimomura, Katsuhiko Nishimori

**Affiliations:** 1 Department of Molecular and Cell Biology, Graduate School of Agricultural Science, Tohoku University, 468-1 Aramaki-Aza-Aoba, Aoba-ku, Sendai, Miyagi 980-0845 Japan; 2 Department of Obesity and Internal Inflammation, Fukushima Medical University, 1 Hikarigaoka, Fukushima, 960-1295 Japan; 3 Department of Bioregulation and Pharmacological Medicine, Fukushima Medical University, 1 Hikarigaoka, Fukushima, 960-1295 Japan; 4 Laboratory for Molecular Neuroscience Medical Research Institute, Tokyo Medical and Dental University, Tokyo, 113-8510 Japan; 5 Innovation Center, Nippon Flour Mills Co., Ltd., 5-1-3 Midorigaoka, Atsugi, Kanagawa 243-0041 Japan; 6 Technology and Development Team for Mouse Phenotype Analysis, Japan Mouse Clinic, RIKEN BioResouce Reserch Center (BRC), 3-1-1 Koyadai, Tsukuba, Ibaraki 305-0074 Japan; 7 Division of Genetic Therapeutics, Center for Medicine, Jichi Medical University, Shimotsuke, Tochigi 329-0498 Japan; 8 Department of Biological Sciences, Louisiana State University, Baton Rouge, LA 70803 USA; 9 Department of Neurochemistry, Graduate School of Medicine, The University of Tokyo, Bunkyo-ku, Tokyo, 113-0033 Japan

**Keywords:** Autism spectrum disorders, Social behaviour

## Abstract

Autism spectrum disorder (ASD) is a continuum of neurodevelopmental disorders and needs new therapeutic approaches. Recently, oxytocin (OXT) showed potential as the first anti-ASD drug. Many reports have described the efficacy of intranasal OXT therapy to improve the core symptoms of patients with ASD; however, the underlying neurobiological mechanism remains unknown. The OXT/oxytocin receptor (OXTR) system, through the lateral septum (LS), contributes to social behavior, which is disrupted in ASD. Therefore, we selectively express hM3Dq in OXTR-expressing (OXTR+) neurons in the LS to investigate this effect in ASD mouse models developed by environmental and genetic cues. In mice that received valproic acid (environmental cue), we demonstrated successful recovery of impaired social memory with three-chamber test after OXTR+ neuron activation in the LS. Application of a similar strategy to *Nl3*^*R451C*^ knock-in mice (genetic cue) also caused successful recovery of impaired social memory in single field test. OXTR+ neurons in the LS, which are activated by social stimuli, are projected to the CA1 region of the hippocampus. This study identified a candidate mechanism for improving core symptoms of ASD by artificial activation of DREADDs, as a simulation of OXT administration to activate OXTR+ neurons in the LS.

## Introduction

Autism spectrum disorder (ASD) is a heritable neurodevelopmental disorder associated with a combination of heterogeneous genetic variations; however, environmental cues have also been suspected to be associated with ASD. The core symptoms of ASD are social deficits, communication difficulties, and repetitive and stereotyped behaviors^1^. Although ASD is estimated to affect approximately 1% of children globally^2^, an effective therapy for ASD remains to be developed. Mouse models of ASD are based either on the administration of chemical agents or on editing the genomic DNA sequence of candidate genes suspected to cause ASD. Both these methods typically result in mice with abnormal behaviors, resembling those observed in patients with ASD. Presently, such animal models are being utilized in the study of the pathophysiological mechanisms of ASD and in the development of novel therapeutic tools and strategies for the treatment of ASD. In utero valproic acid (sodium valproate; VPA) exposure is widely used to induce an animal model of ASD. VPA is a known therapeutic agent to treat psychiatric disorders such as bipolar disorder and epilepsy^3^; however, a higher prevalence of ASD has been observed in children born from mothers who were exposed to VPA during pregnancy^4–7^. Similarly, mice or rats foetuses exposed to VPA had a decrease in the number of Purkinje cells in the intermediate zone of the cerebellum^8^, as well as exhibited repetitive behavior and impaired social behaviors^9^. These observations suggest that VPA-induced rodent models of ASD may be helpful to study the relationship between impaired behaviors and neuronal pathology associated with ASD. However, several ASD mouse models harbour gene mutations suspected to cause ASD, such as neuroligin3 (R451C) knock-in (*Nl3*^*R451C*^) mice. The *Nl3*^*R451C*^ mouse model with a mutation of *Nl3*^10^ is one of representative gene-modified ASD mouse models. An X-linked point mutation leading to an arginine to cysteine substitution at amino acid 451 (R451C) of NL3 (NL3R451C) has been identified in a subset of human patients with ASD^11^. NL3 is a postsynaptic cell-adhesion molecule that acts as a ligand for neurexins and regulates synaptogenesis^12^. Knock-in mice expressing the Nl3R451C imitated NL3R451C point mutation and showed impaired social interaction and excitatory/inhibitory (E/I) imbalance^10^. Several single nucleotide polymorphisms in the oxytocin receptor (*Oxtr*) gene have been reported that are linked to impairment of socio-cognitive function^13^, and *Oxtr* is considered to be an ASD-causative gene. OXTR is a G-protein coupled receptor (GPCR) with seven transmembrane domains. In the rodent brain, expression of OXTR is found in the olfactory pathway, olfactory bulb, and accessory olfactory nucleus, and as a processing pathway for social information, the medial amygdala, central amygdala, bed nucleus of stria terminalis, piriform cortex, lateral septum (LS), hippocampus, nucleus accumbens, and prefrontal cortex. In the primate, OXTR expression is detected in the visual processing area, superior colliculus, superficial gray layer of the superior colliculus, pulvinar, and primary visual cortex. In addition, OXTR expression is found in the cholinergic region including the nucleus basalis of Meynert and pedunculopontine tegmental nucleus, which are known to regulate selective attention and motivation^14^. Oxytocin (OXT) is one of two neurohypophysial nonapeptide hormones synthesized in the paraventricular nucleus (PVN) and supraoptic nucleus and binds to the OXTR to induce the activation of Gq/11, thus eliciting various cellular responses. Such neural excitation in specific regions following the activation of specific neural circuits has been suggested to control sociosexual behaviors, such as pair bonding, social memory, and prosocial behaviors. These behavioral controls are mediated via complex neural circuits, including the neurons expressing OXT or OXTR.

Recent studies have demonstrated that the impaired behaviors observed in patients with ASD and animal models of ASD were recovered to some extent by intranasal administration of OXT; therefore, OXT is now suggested to become an approved therapeutic drug for the treatment of ASD. In view of this evidence, clinical trials to evaluate the effect of OXT on patients with ASD^15–17^ and basic research to assess the effect of OXT in animal models of ASD are now being conducted^18–20^. Previous studies have reported that intranasal administration or intraperitoneal injection of OXT in animal models of ASD resulted in improvements in social memory and other symptoms of ASD^15–17^. It was reported that the central OXT system is impaired in VPA rats. OXT expressing cells and mRNA level were lower in the hypothalamus of VPA rats compared to control rats. Additionally, single intranasal administration of OXT improved the impaired social behavior of adolescent VPA rats. It was also shown that after early neonatal OXT manipulation, the number of OXT-expressing cells was significantly restored in the PVN of VPA rats, and there was a long-term therapeutic effect on autistic-like behavior^21^.

These studies strongly suggest that OXT administration has significant potential in the treatment of ASD. However, the causative gene mutations or environmental factors, other than *Oxt* or *Oxtr* gene mutations, neural mechanism of ASD, and therapeutic mechanism underlying OXT-induced improvement of social deficits in these ASD models remain largely unknown. We have previously reported that the LS is an important brain region in the regulation of social memory^22^, and speculated that OXT might contribute to the amelioration of ASD-like behaviors via the LS.

In a psychological study of human, Chen et al. reported the close relation between human social behavior and neural activation after intranasal administration of OXT, in lateral septal nucleus, where the level of *Oxtr* gene expression was suspected to be affected by its DNA methylation level^23^. However, so far there’s no literature with description that the OXTR expressed in lateral septal nucleus in human brain closely relates to the therapeutic mechanism by OXT, intranasally administrated to ASD patients.

OXTR belongs to class 1 family of GPCRs, and it is coupled with trimeric G proteins, whose α subunit is Gαq/11. Stimulation by binding of OXT, the natural ligand to this receptor, facilitates signal exertion by activation of Phospholipase C, followed by increase of intercellular Ca^2+^, activation of Protein kinase C, and multiple cellular reactions. Designer receptors exclusively activated by a designer drug (DREADD) are family of engineered G protein-coupled receptors derived from the human mAChR, which can control three major GPCR signaling pathways (Gαq/11, Gαi, and Gαs)- depending on specific mutations in their structures. DREADD subtype 3 (hM3Dq), which is coupled with Gαq, exerts signals and induces firing of neurons after binding of clozapine N-oxide (CNO) in vitro^24^ and in vivo^25^. It was reported that the effect of CNO was observed 30 min after its administration and continued for up to 120 min^25–27^. Researchers generally believe that major signal pathways of hM3Dq can imitate those of many Gαq type G protein-coupled receptors. In this study, we aimed to investigate the underlying mechanism of the effect of OXTR activation on autistic symptoms in ASD mouse models developed by environmental and genetic cues.

## Results

### Activation of OXTR-expressing neurons in the LS ameliorated the abnormal social novelty and anxiety observed in VPA mice

**Figure 1:**
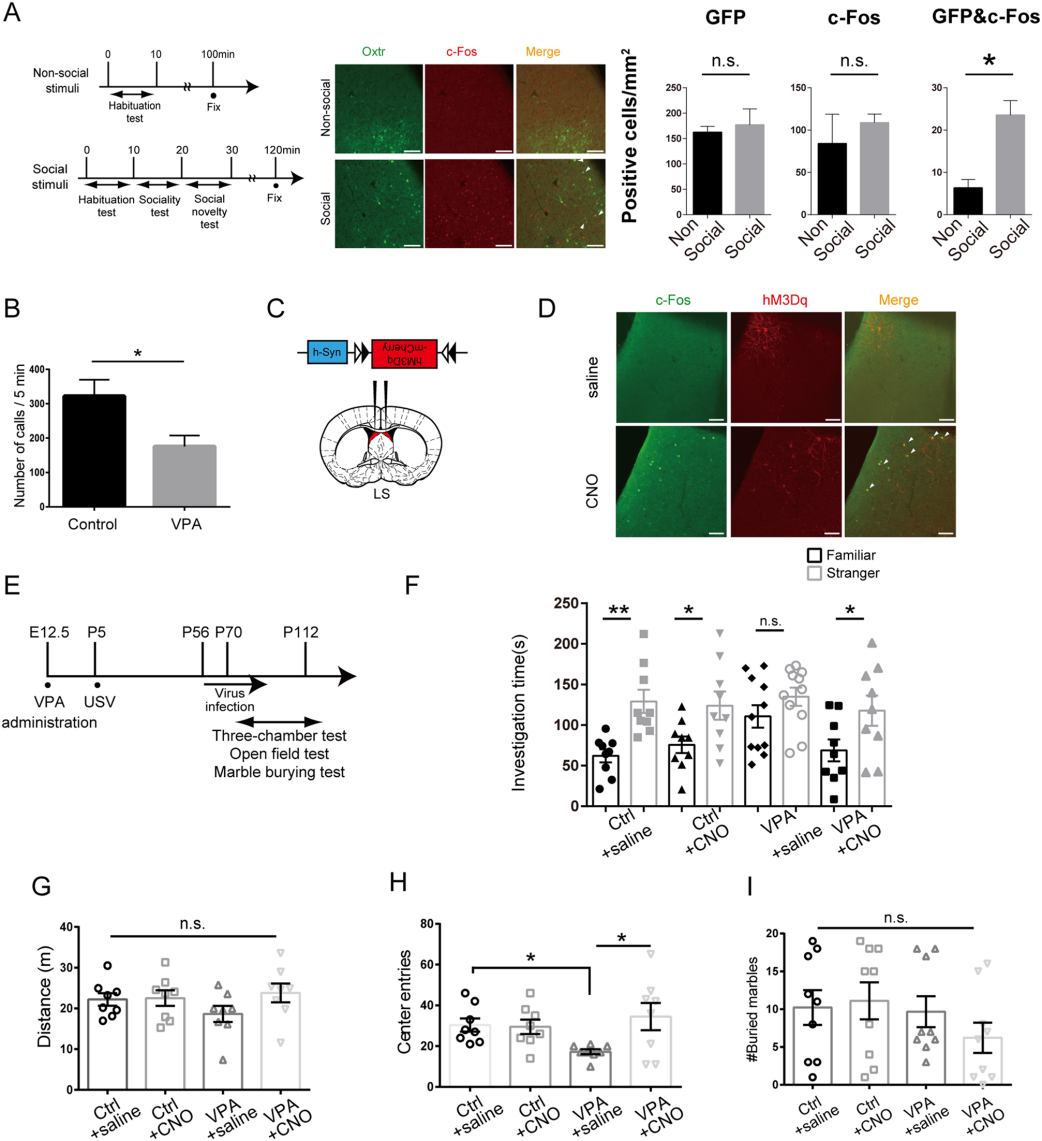
Activation of oxytocin receptor-expressing neurons in the lateral septum (LS) ameliorated the abnormal social novelty observed in mice exposed to valproic acid (VPA) and elicited an anxiolytic effect. (**A**) Schematic diagram of the protocol (left). Confocal images of the LS (immunostained with c-Fos and Venus) in *Oxtr-Venus* mice that received social or non-social stimulation (middle). Number of c-Fos and Venus positive cells and the number of c-Fos (+) and Venus (+) neurons after social novelty test (N = 3) and non-social state in the LS (N = 3). Scale bars indicate 100 µm. (**B**) Mouse communicative function was assessed by recording neonatal ultrasonic vocalizations. Control: N = 10, VPA: N = 23. Control were *Oxtr-Cre* mice treated with saline at E12.5. VPA were *Oxtr-Cre* mice treated with VPA at E12.5. (**C**) AAV vector constructs to express designer receptors exclusively activated by designer drugs (DREADD) (hM3Dq) (top) and a stereotaxic map displaying the location of hM3Dq expression (down). Adapted from Paxinos and Watson “The Mouse Brain in Stereotaxic Coodinate” ^69^. (**D**) hM3Dq was expressed specifically in the LS (including Bregma + 0.26) of *Oxtr-Cre* mice and activated by clozapine N-oxide (CNO). Scale bars indicate 100 µm. (**E**) Schematic diagram of the protocol for the behavioral test administered to VPA mice. (**F**) Investigation time quantified during the social novelty test (third stage). Control (Ctrl) + saline, Ctrl + CNO, VPA + CNO: N = 8 per group; VPA + saline: N = 11. (**G**) The saline- and CNO-treated Ctrl and VPA mice exhibited similar levels of locomotor activity in terms of the total distance traveled as measured by open field test. N = 8 per group. (**H**) Number of center entries quantified by the open field test for the saline- or CNO-treated Ctrl and VPA mice. N = 8 per group. (**I**) Number of marbles buried by the saline- and CNO-treated Ctrl and VPA mice. N = 9 per group. Unpaired *t*-tests were performed for (**A**,**B**). Tukey's test and Dunn’s multiple comparison test were performed for (**G**) and (**H**,**I**). Paired *t*-test was performed for (**F**). Data are expressed as the mean ± standard error of the mean. *,**p < 0.05, 0.01. All the test mice using (**F**–**H**), and (**I**) were infected with hM3Dq vector earlier than 2 weeks.

To examine whether the LS was involved in social novelty, we first observed c-Fos protein expression in the LS of *Oxtr-Venus* (*Oxtr*^*venus/*+^) mice when they were socially or non-socially stimulated. *Oxtr-Venus* mice specifically express the Venus protein in OXTR expressing (OXTR+) neurons. We observed high expression of c-Fos in the socially stimulated condition (Fig. 1A). Based on previous findings^22,28^, we expected that the activation of OXTR+ neurons in the LS and in the neural circuits composed of such neurons, with the administration of OXT might compensate for the impaired OXTR signalling in VPA mice and ameliorate their ASD-like symptoms. We treated pregnant *Oxtr-Cre* (*Oxtr*^*cre/*+^) mice (which specifically express the Cre protein in only the OXTR+ neurons)^29^ with VPA to generate animal models of ASD (VPA mice). We then applied the DREADDs artificial receptor system^24^ to generate a mouse model with restricted activation of neurons in the LS and neuron subtype (OXTR+)-specific manner to enhance OXTR signalling. To conduct the “signal enhancement” experiment, we first confirmed that the VPA mice exhibited autism symptoms after VPA (600 mg/kg) was administered intraperitoneally to their mothers on E12.5. The pups exhibited autism symptoms, indicated by significant reduction in the number of ultrasonic vocalizations, as demonstrated previously (Fig. 1B)^30,31^. To generate a signal similar to that of OXTR (when activated by OXT), we used the artificial receptor, hM3Dq, which is a Gq/11-coupled receptor, in the DREADDs experiment (Fig. 1C). We injected an Adeno-associated virus (AAV)-DIO-hM3Dq-mCherry vector (hM3Dq vector) into the LS of *Oxtr-Cre* mice (healthy controls), checked for sufficient transfection by the AAV vector, and confirmed activation of the infected neurons by administering CNO to the AAV-transfected mice (Fig. 1D). Similarly, an hM3Dq vector was injected into the LS of VPA mice; subsequently, the operated mice were characterized according to social novelty (using the three-chamber test), anxiety (using the open-field test), and repetitive behaviors (using the marble burying test), which are considered related to dysfunction in human ASD (Fig. 1E). Saline-treated VPA mice showed abnormal social novelty, whereas CNO treatment of VPA mice transfected with the hM3Dq vector resulted in dramatic improvement in preference for social novelty (Fig. 1F). VPA mice showed normal sociability (Supplemental Figure S1). We subsequently performed behavioral tests for anxiety, followed by tests for repetitive behavior. Although abnormal repetitive behavior was not observed in the open field test (Fig. 1I), contrary to previous reports^32^, we detected high anxiety in the VPA mice, which was ameliorated after activation of OXTR+ neurons (Fig. 1B). Importantly, we observed no change in total locomotor activity (Fig. 1G).

### Activation of OXTR-expressing neurons in the LS ameliorated abnormal social novelty in *Nl3*^*R451C*^ mice

**Figure 2:**
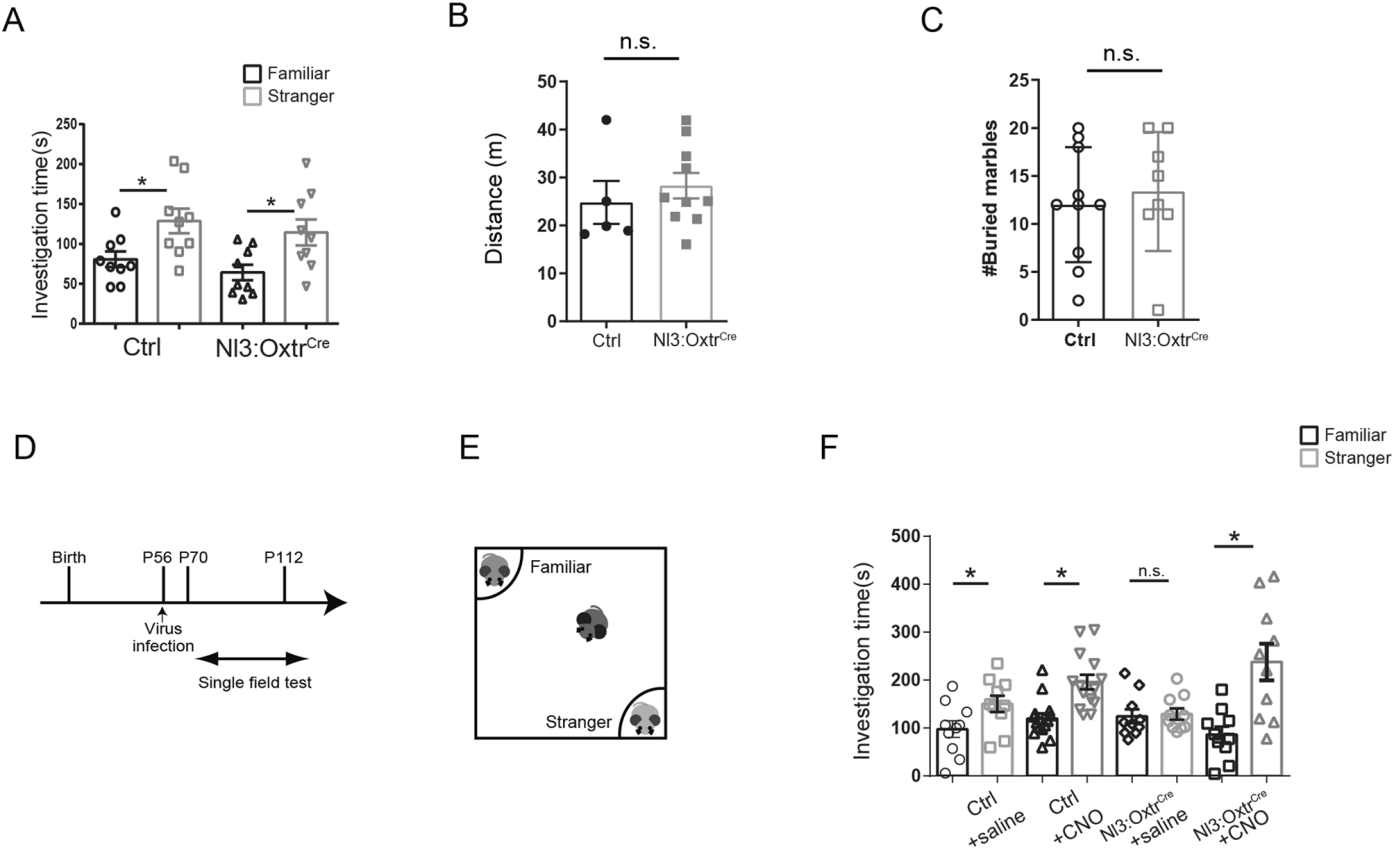
Activation of oxytocin receptor-expressing neurons in the lateral septum ameliorated the abnormal social novelty in *Nl3*^*R451C*^*: Oxtr*^cre/+^ mice. (**A**) Investigation time quantified during the social novelty by three-chamber test (third stage) of *Oxtr-Cre* (Ctrl) and *Nl3*^*R451C*^*: Oxtr*^*cre/*+^ mice (*Nl3:Oxtr*^*cre/*+^). N = 9 per group. (**B**) Total distance traveled as measured with open field test. Ctrl: N = 5, *Nl3:Oxtr*^*cre/*+^: N = 10. (**C**) Number of buried marbles quantified repetitive behavior with the marble burying test. Ctrl: N = 10, *Nl3:Oxtr*^*cre/*+^: N = 8. (**D**) Schematic diagram of the protocol for virus infection and the single field test in *Nl3:Oxtr*^*cre/*+^ mice. (**E**) Behavioral schematic of the single field test. (**F**) Investigation time during the social novelty (third stage) by single field test, administered saline to Ctrl (N = 10), CNO to Ctrl (N = 15), saline to *Nl3:Oxtr*^*cre/*+^ (N = 10) and CNO to *Nl3:Oxtr*^*cre/*+^ (N = 10). All mice using the single field test were infected with hM3Dq vector earlier than 2 weeks. Paired *t*-tests were performed for three-chamber test and single field test (**A**,**F**) and Mann–Whitney tests were performed for open field test and marble burying test (**B**,**C**). *^,^**p < 0.05, 0.01.

Our “signal enhancement” experiment for OXTR+ neurons in the LS of VPA mice implied that the LS might be an important target for OXT-mediated therapeutic effects in ASD mice. We next examined whether the same strategy would be effective in a genetic mouse model of ASD, *Nl3*^*R451C*^ mice, generated by mutation of the *Nl3* gene. We tested the social novelty of the *Nl3*^*R451C*^*: Oxtr-Cre* (*Nl3:Oxtr*^*Cre/*+^) mice using the three-chamber test and found no abnormal social novelty (Fig. 2A). Abnormal sociability was also not found in *Nl3:Oxtr*^*Cre/*+^ mice (Supplemental Figure S2). Furthermore, we tested locomotion and assessed repetitive behavior in these mice, using the open field test and marble burying test respectively. Both behaviors were normal relative to those of the wild-type mice (Fig. 2B,C). We next examined social novelty using the “social discrimination test,” wherein a “single field” was utilized instead of three-chambers^33^ (Fig. 2F). We detected impaired preference for social novelty in *Nl3:Oxtr*^*Cre/*+^ mice; this impairment was ameliorated by the administration of CNO in AAV vector-transfected mice (Fig. 2F). *Nl3:Oxtr*^*Cre/*+^ mice showed normal sociability in the single field test (Supplemental Figure S2).

### OXTR-expressing neurons in the hippocampal CA1 region projected from the LS are activated by social stimuli

**Figure 3:**
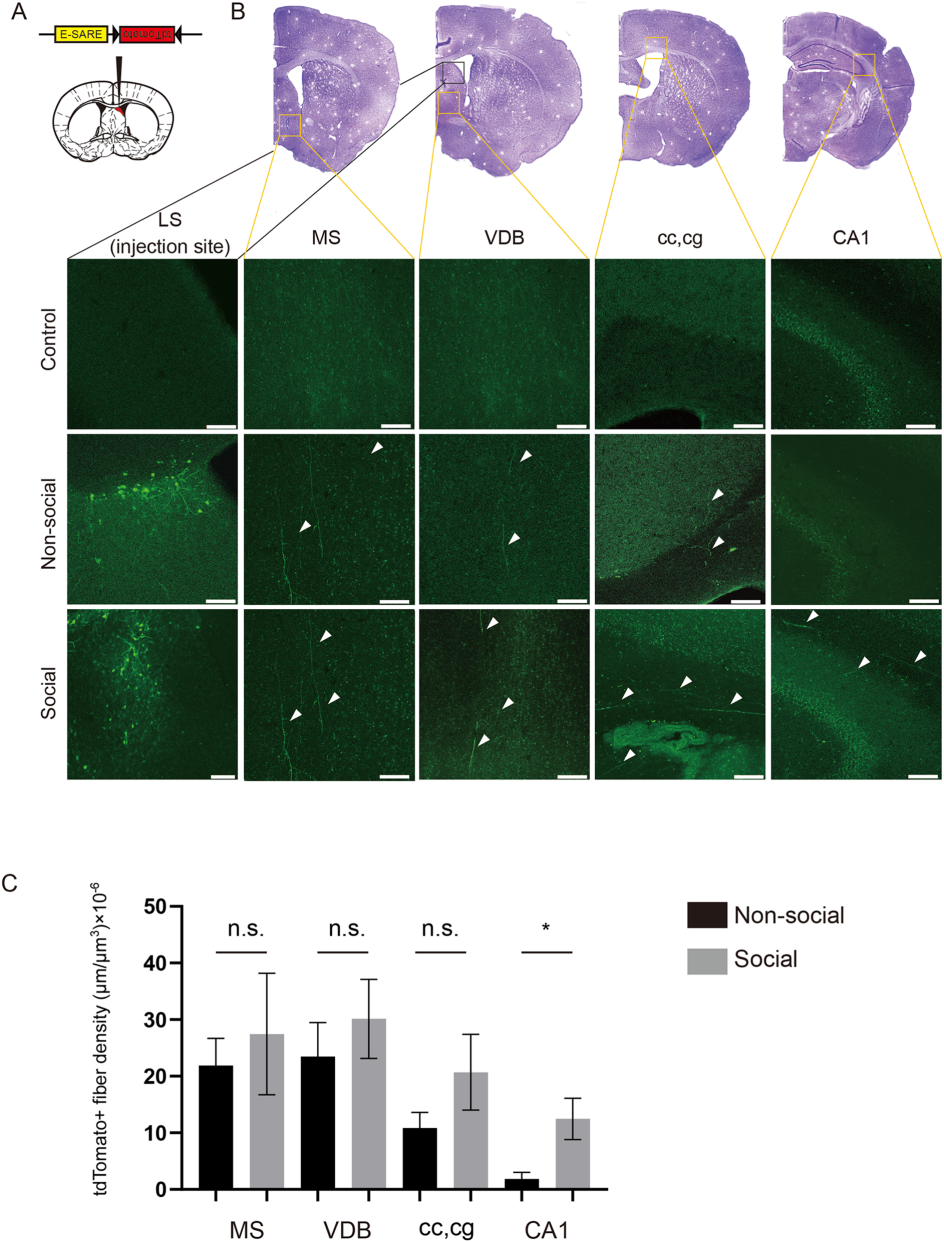
Oxytocin receptor (OXTR)-expressing neurons in the lateral septum (LS), which were activated by social stimulation, projected to the CA1 region of the hippocampus. (**A**) AAV vector constructs expressing E-SARE-FLEX-tdTomato (left) and a stereotaxic map displaying the location of vector expression (right). Brain illustration is an adaption as well as Fig. 1C. (**B**) Projection site of the OXTR+ neurons in the LS that were activated by social or non-social stimulation. (**C**) Quantitative analysis of the density of social stimuli-activated axon projection was performed as indicated in the “Methods”. Scale bars indicate 100 µm. (Control, no viral infection and stimulation; *MS* medial septum, *VDB* nucleus of the vertical limb of the diagonal band, *cc* corpus callosum, *cg* cingulum) AAV-E-SARE-FLEX-tdTomato was used for the green color in pseudo coloring. N = 3 (Social), N = 6 (Non-social).

**Figure 4:**
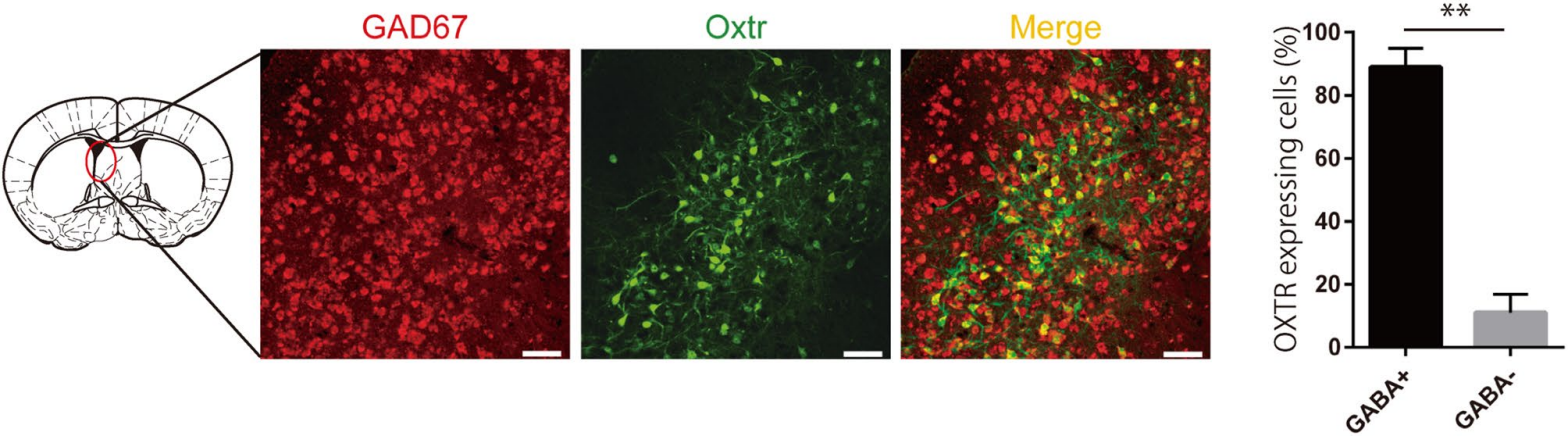
Oxytocin receptor (OXTR)-expressing neurons in the lateral septum (LS) were GABAergic neurons. Confocal images of the LS show neurons expressing mRNAs for GAD67 and OXTR in *Oxtr-Venus* mice (left). The ratio of the number of OXTR+ neurons of GABA (+) cells and GABA (−) cells in the LS is shown (N = 3) (right). Unpaired t-tests were performed for the graph. ** *p* < 0.01. The brain illustration is an adaption as well as Figs. 1C and 3A.

As illustrated in Figs. 1 and 2, artificial activation of OXTR+ neurons in the LS improved preference for social novelty in VPA-treated mice and *Nl3:Oxtr*^*Cre/*+^ mice. To further investigate the mechanism underlying this improvement, we attempted to obtain anatomical data to identify the regions projected by the OXTR+ neurons in the LS and whose neural activities are regulated to control preference for social novelty. Kawashima et al. described a synthetically enhanced synaptic activity-responsive element (E-SARE) of Arc, one of the immediate early genes^34^, which enables selective labelling of neurons that respond to particular stimuli. We designed a “FLEX” type AAV vector with the E-SARE and tdTomato reporter cDNA. The resultant AAV-E-SARE-FLEX-tdTomato was injected into the LS region of *Oxtr-Cre* mice (Fig. 3A), and the infected mice were exposed to social stimuli (Social). Analysis of the projections from the OXTR+ neurons in the LS activated by social stimuli revealed projections to the medial septum (MS), nucleus of the vertical limb of the diagonal band (VDB), corpus callosum (cc), cingulum (cg), and CA1 region of the hippocampus. Moreover, projections to the MS, VDB, cc, and cg were observed when the transfected mice were non-socially stimulated (Non-Social); however, projections from the LS to CA1 were hardly detected in non-socially stimulated mice (Fig. 3B). Fiber density was calculated by the ratio of total fiber length/volume of the reference region (Fig. 3C)^35^. The total length of activated OXTR+ neurons projected from LS to CA1 in social test was significantly larger than in non-social test, but this difference was not obtainable in the MS, VDB, and cc, and cg (Fig. 3C). We then confirmed the co-localization of *Gad67* mRNA and OXTR using the *Oxtr-Venus* mice to examine the OXTR+ neurons in the LS. The fluorescent signal for > 90% of the OXTR+ neurons in the LS co-localized with that of *Gad67* (Fig. 4). The gene expression levels of *Oxtr, Gad65,* and *Gad67* in the LS of VPA mice and *Nl3:Oxtr*^*Cre/*+^ mice, assessed by quantitative real-time PCR, did not change compared with those in control mice (Supplemental Figure S3).

These data signify the importance of the suppressive projections from OXTR+ neurons in the LS to the CA1 in mediating preference for social novelty.

## Discussion

In the present study, we analysed the neuropharmacological mechanism in a neurons-subtype and nuclei specific manners, underlying the amelioration of autism symptoms following OXT administration using the “signal enhancement strategy”. This strategy was chosen on the basis that the physiological effects of OXT in the brain are principally generated by the binding of OXT to particular neurons expressing OXTR, and on the presumption that OXTR is a physiologically major or unique receptor for OXT in brain, and used a Gq/11 type DREADD signal, similar to OXTR, generated in the OXTR+ neurons of specific brain regions. We selected the LS as the target region in mouse models with ASD because previous studies have identified a strong association of this region with social novelty^22^. Few reports have stated that the OXT/OXTR signalling in the LS influences social behavior in normal rodents, but not in ASD model rodents. In the LS of male rats, OXT release was increased during retrieval of social memory for male juvenile stimuli, and administration of OXTR-antagonist into the LS impaired the retrieval of social memory in both male juveniles and female adult rats^36^. Local OXT release within LS of male rats occurred in response to social stimuli or exposure to emotional stress such as social defeat^37,38^. On the other hand, the experience of social fear conditioning blocks OXT release within the LS during extinction^39^. Moreover, administration of OXT or enhancement of OXTR expression in the GABAergic neurons in the LS of virgin mice attenuated their social fear^40^.

We have already reported that LS-region specific deletion of *Oxtr* gene, using *Oxtr*^*fx/fx*^ mice combined with injection of AAV-Cre, impaired social novelty, as evaluated with the three-chamber test^22^. The administration of arginine vasopressin, a type of neuro-hypophysial hormone closely related to oxytocin, to the LS of rat brain rescued impaired social memory^41^.

There are multiple reports about the behavioral function and expressed molecules in LS of Prairie Vole (Microtus ochrogaster). In the LS of Prairie Vole, there is condensed localization of OXTR and its closely related family member molecule, the vasopressin receptor 1a, which has a critical role in pair bonding behavior^42,43^ and other social/prosocial behaviors^44^. The deletion of the *Oxtr* gene in Prairie Voles caused impaired social novelty in the three-chamber test^45^. The combination of pre-and post-wean socially depleted environments in Prairie Voles reduced social discrimination and socio-spatial memory, and OXTR binding density showed an increase in LS^44^. Adult male rats underwent social instability stress in adolescence (SS rats) and impaired social interaction and social recognition. OXTR binding density of the SS rat brain increased in the LS and nACC (nucleus accumbens shell)^46^.

Although our study has not proved that the suppression of OXTR expressed in LS was the major cause for impaired social memory in *Oxtr* gene knockout (KO) prairie vole, we suspected that the lack of oxytocin/oxytocin receptor signal in LS might be critical factor to cause impairment in their social novelty.

As a result of the signal enhancement strategy, using artificial expression and activation of hM3Dq molecule in OXTR+ neurons in the LS, we found that the LS could be a target for improvement of ASD symptoms by administration of OXT in at least two mouse models of ASD. In this study, we selected LS as the target region. However, studies have identified several brain regions associated with ASD. One of them is the amygdala, a well-known brain region with critical functions in emotion, fear, and memory; brain imaging revealed an increased volume of amygdala in patients with ASD compared healthy controls^47,48^. Therefore, we deduced that enhanced signalling via the OXTR+ neurons in the amygdala in addition to the LS contributed to the amelioration of ASD symptoms. Additionally, we have obtained similar data, which shows that enhanced signalling in the OXTR+ neurons of the medial amygdala ameliorated ASD symptoms (manuscript in preparation). Though it’s our final goal to solve the complex neurocircuits for formation of social novelty, which are composed of both the inhibitory neurons, expressing OXTR in lateral septal nucleus, shown in the present work, and the excitatory neurons, also expressing OXTR, in medial amygdaloid nucleus (data not shown), here we report simply about neurons expressing OXTR in LS. Figure 4 shows that > 90% of the OXTR+ neurons in the LS were GABAergic neurons. Based on these findings, we conclude that the inhibitory signals in the LS induced by the “signal enhancement,” which when used in combination, contributed to improve social novelty in VPA mice and *Nl3:Oxtr*^*Cre/*+^mice.

As illustrated in Figs. 1 and 2, activation of OXTR+ neurons in the LS recovered the preference for social novelty in two mouse models of ASD that were generated to respond to an environmental cue (VPA mice) or a genetic mutation of *Nl3*^*R451C*^ mice which is one of the representative ASD model with a genetical cue. Previous studies that used VPA-induced animal models of ASD have reported decreased activity of the GABAergic interneurons^49^ and increased activity of the glutamatergic neurons and glutamate receptor^50–52^; these results suggest an excitatory/inhibitory imbalance in the neural circuit with an excitation-dominant condition in VPA-induced animal models. Activated OXTR+ neurons via DREADD system in LS of *Nl3:Oxtr*^*Cre/*+^ resulted in the improvement of preference for social novelty as observed with the single field test, but not with the three-chamber test. We consider the possibility that *Nl3:Oxtr*^*Cre/*+^ mice have additional impairment in behavior, other than their abnormality in social novelty. This impairment is suspected to be highly sensitive to the complicated structure with larger size of the three-chamber arena, which has three rooms divided each other, compared with the apparatus for single field test. For example, there is a difference with respect to the ease of detection of an olfactory stimulus. All neuronal cells in the olfactory bulb express Nl3^53^, and *Nl3* KO mice show olfactory deficiency^12^. As there are two walls in the apparatus for three-chamber and the size of the apparatus for the test is larger than for single field, the spreading of the smell of the stimulant may be different for both tests. We did not detect any repetitive behavior in VPA mice (Fig. 1I). It has been reported that VPA-treated C57BL/6 mice demonstrated repetitive behavior, which was evaluated with the Marble bury test^32^. On the other hand, *Oxtr-Cre* mice line, which was used in the present study after treatment with VPA in utero, has 129× C57BL/6J mixed genetic background. This difference in the genetic background may be responsible for inability to detect repetitive behavior of VPA-treated ASD model mice.

We showed the OXTR+ neurons, activated by social stimulation in the LS, may project to the CA1 region of the hippocampus (Fig. 3). Various reports have described the function of the hippocampus in the formation of social memory^33,54,55^. The hippocampus is composed of several sub-regions and is essential for social memory. In the neural circuit of social memory, information proceeds from the entorhinal cortex to the dentate gyrus, CA3, and then to CA1, which is the main hippocampal output region^55–57^. The CA2 region is also an important hub for the formation of social memory^55^. The hippocampus is composed of several subregions and is essential for social memory. As most of the OXTR+ neurons in the LS are GABAergic (Fig. 4), we suspected that a portion of those neurons projecting to the CA1 likely modulate social novelty by altering the E/I balance in the CA1, such as through inhibition of interneurons and resultant disinhibition of principal neurons in the CA1^58^. This would lead to the activation of *N-*methyl-d-aspartate (NMDA) receptor-mediated functions in the hippocampus, including establishing social memory. On the other hand, suppressed expression of GAD65 or GAD67 (which are the GABA-synthetic enzymes) were observed in multiple regions including the hippocampus^59^ in the brains of VPA mice. We suspected that these suppressed activities of GABAergic neurons might subsequently cause abnormal social novelty due to impaired signal transmission in the hippocampus. In our study, with application of DREADDs to the LS of VPA mice, we induced artificial activation of OXTR+ neurons to further suppress basket cells in the hippocampus which might be one of the projected targets of OXTR+ neurons from the LS (Fig. 4). We speculated that the resultant disinhibition of principal neurons in CA1 might increase the output signal required for social memory, leading to amelioration of social novelty. On the other hand, the PV basket cells in hippocampal CA1 region of *Nl3*^*R451C*^ mice formed disrupted tonic endocannabinoid signaling, with notable reduction of IPSC^60^, while cholecystokinin basket cells suffered only a slight impairment of IPSC in the similar condition. Our finding that the artificial activation of septohippocampal projections from OXTR+ GABAergic neurons in LS in the *Nl3*^*R451C*^ mouse brain using DREADDs system, might cause a compensatory effect on impaired function of CA1 basket cells. This may lead to normal disinhibition of pyramidal neurons in CA1, followed by establishment of normal social memory. As we have not yet identified the subtype of basket cells, to which social novelty related OXTR+ septohippocampal neurons project, the detailed mechanism that explains this hypothesis should be explored in further experiments including anatomical and electrophysiological studies.

We indicated the suppressive function of activation of OXTR+ neurons in the LS on anxiety behavior (Fig. 1H). This result shows the anxiolytic potential of activation of OXTR+ neurons in the LS. Several studies have reported the anxiolytic effect of OXT in mice LS. OXT/OXTR signalling in the LS region enhanced fear memory after treatment with social defeat demonstrated the conditional gene knockout of *Oxtr* in the LS by injection of AAV-Cre to the LS of *Oxtr*^fx/fx^ mice^61,62^. In contrast, when these mice had social buffering treatment, the OXT/OXTR signal had an enhancing effect on reducing fear memory. The lactating mice showed activation of OXT system in the LS and did not express social fear and inhibition of OXT signalling in the LS of Lactating mice reinstated social fear^40^.

It has been reported that activation of LS-projecting ventral hippocampal neurons caused subsequent suppression of anxiety behavior^63^. Because most projecting neurons in the hippocampus are glutamatergic, we assumed that anxiety might be suppressed via the activation of inhibitory neurons, including the OXTR+ neurons in the LS.

Although we could not show the signals induced by OXTR to be totally identical to those induced by hM3Dq, we detected that the major signalling pathways of OXTR are similar to those of hM3Dq. Herein, we demonstrate that OXTR+ neurons in the LS are important targets of OXT. Additionally, our findings suggest that the same region might function as the target of OXT in similar related disorders. It is not known whether OXT is applicable to all types of patients with ASD. With the acceleration in the application of this analytical approach to multiple mouse models of ASD with various cues, we will potentially obtain further insight to expand “OXT treatment” therapy as a more efficient and effective strategy to treat many patients with ASD.

## Methods

### Animals

*Oxtr-Venus* mice, *Oxtr-Cre* mice, and *Nl3*^*R451C*^ mice were generated as previously described^10,29,64^. Genetic background of *Oxtr-Cre* were 129× C57BL/6J^29^ and *Nl3*^*R451C*^ were Sv129/C57Bl6^10^. On embryonic day (E12.5), pregnant *Oxtr-Cre* mice received a single i.p. injection of 600 mg/kg VPA (Tokyo Chemical Industry co) dissolved in saline (VPA mice) or only saline (control mice). After weaning at 21 days of age, the mice were allowed ad libitum access to standard chow and water, and kept at 25 °C in a room with a 12 h light/dark cycle. All of the animal protocols and procedures for experiments were approved by the Animal Committee of the Graduate School of Agricultural Science, Tohoku University Ethics Committee, or by Animal Experiments Committee of Fukushima Medical University. All experiments were conducted according to the ethical and safety guidelines of Tohoku University or under the ethical and safety guidelines and regulations of Fukushima Medical University.

### Viral injection

We constructed AAV-E-SARE-FLEX-tdTomato to insert FLEX-eSARE-tdTomato fragment at BamHI and EcoRI site in AAV-MCS (Addgene). The recombinant vectors were packaged by the viral vector core at Jichi Medical University. The viral vector, AAV-hSyn-FLEX-hM3Dq-mCherry (Addgene), was injected bilaterally into the LS (Bregma+ 0.26 mm, lateral+ 0.5 mm, deep+ 2.75 mm) and the viral vector, AAV-E-SARE-FLEX-tdTomato^34^, was injected into one side of the LS on 2 weeks before three-chamber test. The injection volume was 1.0 µl per side.

### Drugs

The animals were injected intraperitoneally with 300 μg/kg CNO (Sigma-Aldrich) 30 min before each behavioral test.

### Ultrasonic vocalization analysis (USV test)

Pups were isolated from their mother on P5 to induce ultrasonic vocalizations. Each pup was placed in a sound-proof chamber (Metris smart chamber, Metris), and ultrasonic vocalizations were recorded for 5 min. The vocalizations were analyzed using Sonotrack (Metris). Any frequency from 25 to 100 kHz was counted as a pup vocalization.

### Behavioral tests

All behavioral experiments were conducted during the light phase (9 a.m. to 7 p.m.) for male mice aged 10–16 weeks. Animals were habituated to the testing room for at least 1 day before any experiment. For behavior test using DREADD system, littermates were separated into two groups at random. Littermates belonging to one group were administered with saline and the other with CNO 30 min before the behavior test.

### Three-chamber test

The test was performed as previously described in literature^65^. In brief, to assess sociability and social novelty, we used a three-compartment social behavior chamber. The chamber was a polyvinyl chloride box (63 cm long × 41 cm wide × 30 cm tall) divided into three compartments with walls made from clear Plexiglas, with small rectangular openings (5 cm wide × 3 cm tall) allowing access into each chamber. A wire cage (10 cm bottom diameter, 18 cm tall, vertical bars 1 cm apart) was placed in each side compartment. The wire cage allowed for auditory, visual, olfactory interactions, and nose contact between the wire bars, but prevented fighting, between experimental and stimulus mice. The test consisted of 3 × 10 min sessions. The first session was habituation, in which no stimuli were present. After habituation, the second session was sociability, in which a stimulus mouse (familiar mouse) and an empty cage were present in separate chambers. Adult DBA/2J male mice were selected to be stimulus mice due to their relatively mild characteristics^65^. The last session measured preference for social novelty, in which the novel DBA/2J mouse (unfamiliar mouse) was placed inside the remaining (empty) cage. The test mouse thus had a choice between the familiar mouse and the novel mouse. DBA/2J mice were two to four months old. Each mouse was purchased from a different breeder; one is from CLEA Japan (Tokyo, Japan) and the other is from Japan SLC (Fukuoka, Japan). The amount of time spent in each contact zone, defined as the area within 5 cm from the wire cage, was recorded by a video camera fitted on top of the box. Data acquisition and analysis were performed automatically with the use of ANY-maze behavior tracking software (Stoelting).

### Marble burying test

The marble burying test was performed as previously described^66^. Briefly, before the tests, the mice were acclimated to the test cage (28 × 19 × 30 cm) laid with clean wood chips to a depth of 5 cm and illuminated at 500 lux. The mice were then placed in the test cage containing 20 equidistantly arranged blue marbles (14 mm in diameter). We counted the number of marbles obscured by at least two-thirds of the surface 20 min after placing the mice in the test box.

### Open field test

The open field test was performed as previously described^67^. Each mouse was placed in the center of an open arena (41 × 41 × 35 cm) illuminated at 500 lux and allowed to explore freely for 10 min. During the tests, their position was continually monitored using ANY-maze software (Stoelting). The total distance travelled and the number of entries to the central area (20 × 20 cm) were assessed.

### Single field test

The single field test was performed as previously reported^68^, in a 41 × 41 × 35 cm white plastic box illuminated at 10 lux. A wire cage (10 cm bottom diameter, 18 cm tall, vertical bars 1 cm apart) was placed symmetrically (Fig. 2E). The contact zone was defined to be around 5 cm from the wire cage. The test consisted of the three 10 min sessions. During all the sessions, the test mice could move freely in the box. After each session, mice were taken out of the box. Immediately after the next stimulants had settled down, the test mice were returned to the test arena, and the next session was started. The first session was habituation: the subject mouse was placed in an open arena with two round wire cages, as described above, on opposing corners of the arena. The second session was sociability: an unfamiliar male mouse (stranger 1) that had never been in contact with the subject mouse was placed in one wire cage while the other wire cage was empty. The third session measured preference for social novelty: another unfamiliar male mouse (stranger 2) was placed in the previously empty wire cage. The test mouse could move within the arena freely in each session. The amount of time spent in each contact zone was recorded using a video camera fitted on top of the box, and data acquisition and analysis were performed automatically with the use of ANY-maze software (Stoelting).

### Stimulation

Our “social stimuli” refers to undergoing the social novelty stage (third stage of the three-chamber test) and “non-social stimuli” refers to undergoing the habituation stage (first stage of the three-chamber test). And then, 90 min after the stimulation, the test mice were perfused as follows.

### Immunohistochemistry and image acquisition

The mice were transcardially perfused with saline followed by 4% paraformaldehyde. After perfusion, the brains were extracted and post-fixed in 4% paraformaldehyde for 1 day. The brains were sectioned coronally (50 μm sections) using a vibratome (Leica) and collected in phosphate-buffered saline (PBS). The sections were permeabilized using 0.3% triton/PBS (PBST) for 30 min and then blocked with 1% blocking reagent in maleic acid buffer (Roche) for 30 min. After blocking, the sections were incubated overnight in rabbit polyclonal anti-cFos antibody (1:1000; RPCA-cFos; EnCor) or chicken polyclonal anti-GFP antibody (1:1000; ab13970; Abcam). After incubation with the primary antibody, the sections were washed three times with PBS and incubated with Alexa 594-conjugated donkey anti-rabbit (1:1000; A21207; Life technologies) or Alexa 488-conjugated goat anti-chicken (1:1000; A11039; Life technologies) secondary antibodies and DAPI for 2 h. After incubation with the secondary antibody, the sections were washed two times with PBS. The confocal images were acquired with an LSM780 or 800 (Zeiss) using 10 × or 20 × objective lenses. Three brain slices from each mouse were collected from the bregma (+ 0.4 to + 0.25).

### Analysis of tractus neural projection

Post-fixation, the brains were sectioned coronally (50 μm sections) using a vibratome (Leica) and collected in PBS. The confocal images were acquired with an LSM780 or 800 (Zeiss) using 10 × objective lenses. The fibrous fluorescent signal was defined as activating neuronal cells projected from the LS area. To examine the total length of signal, three sections from all individual mice were analysed. Signal length was measured using ImageJ freeware (National Institute of Health, Bethesda, MD). The relative value indicating the density of axon projection was calculated as follows: total axon length detected by fluorescence in a constant-volume brain cube (50 µm × 870 µm × 650 µm) section from each nucleus in the stimulus condition and those with no stimuli as control were measured, and these data were divided by cube volume and shown^35^.

### In situ hybridization (ISH)

The brains were sectioned coronally (30 μm sections) using a cryostat (Leica). ISH was carried out at room temperature unless otherwise indicated. Brain sections were incubated 10 min in 4% PFA/PB and then washed 3 × 5 min in DEPC-PBS. They were placed in Detergent mix (1% Nonidet P-40, 1% SDS, 0.5% Deoxycholate, 50 mM Tris–HCl (pH 8.0), 1 mM EDTA (pH 8.0), 150 mM NaCl, DEPC-H_2_O) for 2 × 30 min. and then washed 10 min in DEPC-PBS. They were hybridized by incubating in hybridization buffer containing probes for GAD67 (DIG-labeled) (1.0 µg/mL) at 70 °C overnight. Post-hybridization washes were performed sequentially 3 × 45 min at 70 °C in Solution X (2 × SSC, 50% Formamide, 1% SDS, DEPC-H_2_O), 3 × 15 min in wash buffer containing 0.1% Tween-20 in TBS. They were incubated for 1 h in blocking buffer (10% normal sheep serum in TBST). After blocking, they were incubated for 2 h in anti-digoxygen-AP (roche), washed 3 × 15 min in TBST and then incubated 10 min in 0.1 M Tris (pH 8.2). They were incubated 3–4 h in Fast red (roche) and then washed 3 × 10 min in PBS. After washing they were conducted immunohistochemistry as detailed above. Rabbit polyclonal anti-GFP antibody (1:1000; 598; MBL) and Alexa 488-conjugated goat anti-rabbit (1:1000; A11034; Life technologies) were used as primary and secondary antibodies, respectively. Three brain slices from each mouse were collected from Bregma + 0.4 to + 0.25.

### Quantification-real time PCR

Total RNA was extracted from the LS of the test mice by homogenization in RNAiso (Takara #9108, Japan) according to the manufacturer’s instructions. RNA (1 µg) was reverse-transcribed to cDNA using a PrimeScript RT reagent kit with a genomic DNA eraser (Takara #RR047A, Japan) following the manufacturer’s instructions. cDNA concentration was measured using a NanoDrop™ 1000 spectrophotometer (Thermo Fisher Scientific, USA). SYBR Premix Ex Taq II (Takara #RR420A, Japan), and the Thermal Cycler Dice Real Time System (Takara #TP900, Japan) was used for quantitative real-time PCR (qRT-PCR) to detect gene expression. The primers used for target detection were 5′-GCGGGAGCGGATCCTAATA-3′ and 5′-TGGTGCATCCATGGGCTAC-3′ for *Gad1*gene expression, 5′-TCGGAAACACAAGTGGAAGC-3′ and 5′-GACCAGGAGAGCCGAACATT-3′ for *Gad2* gene expression, 5′-GGAAGTCCAGTGGGATGAGA-3′ and 5′-TCCAGCTCCAAATGCTTTCT-3′ for *Oxtr* gene expression, and 5′-TGACGTGCCGCCTGGAGAAA-3′ and 5′-AGTGTAGCCCAAGATGCCCTTCAG-3′ for *Gapdh* gene expression.

### Statistics

Statistical analysis was performed using GraphPad Prism 6 for Windows (GraphPad Software). Statistical comparisons of open field test, self-grooming test and marble burying test for the VPA mice and control mice were performed using one-way ANOVA followed by Tukey's test or Dunn’s multiple comparison test, and paired *t*-test was used to compare three-chamber test and single field test. For *Nl3*^*R451C*^ mice and the controls, statistical comparisons of open field test and marble burying test were performed with Mann–Whitney test, and paired *t*-test was used to compare three-chamber test and single field test. Statistical analysis of the number of stained cells, USV calls, and rate of OXTR+ cells were performed with unpaired *t*-test. The results were considered statistically significant at *p* < 0.05.

## Supplementary Information

Supplementary Figures.
