# Supplementary material for: Targeting oxytocin receptor (Oxtr)-expressing neurons in the lateral septum to restore social novelty in autism spectrum disorder mouse models
Source: Sci Rep. 2020 Dec 17;10:22173. doi: 10.1038/s41598-020-79109-0 (PMC7746761; doi:10.1038/s41598-020-79109-0)

Title

Targeting oxytocin receptor (Oxtr)-expressing neurons in the lateral septum to restore social novelty in autism spectrum disorder mouse model

Author names

Machi Horiai, Ayano Otsuka, Shizu Hidema, Yuichi Hiraoka, Ryotaro Hayashi, Shinji Miyazaki, Tamio Furuse, Hiroaki Mizukami, Ryoichi Teruyama, Masaru Tamura, Haruhiko Bito, Yuko Maejima, Kenju Shimomura, Katsuhiko Nishimori*

Legends of supplementary figures

Supplementary figure 1

Investigation time quantified during the sociability test (2nd stage) by using the three-chamber test. Control (Ctrl)+saline, Ctrl+CNO, VPA+CNO: N = 8 per group; VPA+saline: N = 11.

Supplementary figure 2

(A) Investigation time quantified during the sociability test (2nd stage) by using the three-chamber test of Oxtr-Cre (Ctrl) and $Nl3^{R451C}$ KI: *Oxtr*-Cre ($Nl3:Oxtr^{Cre}$) mice. N=9 per group.
(B) Investigation time quantified during the sociability test (2nd stage) by using the single-field test. Ctrl+saline: N = 10, Ctrl+CNO: N = 15, $Nl3:Oxtr^{Cre}$+saline: N = 10, $Nl3:Oxtr^{Cre}$+CNO: N = 10.

Supplementary figure 3

Expression levels of *Oxtr* (A, D), *Gad1* (B, E), and *Gad2* (C, F) genes by quantitative real-time PCR. The results of VPA-treated *Oxtr*-Cre mice were A, B, and C, and the results of Nl3:Oxtr^{Cre} mice were D, E, and F. Control mice (Ctrl) were *Oxtr*-Cre. N=6 per group

Supplementary figure 1

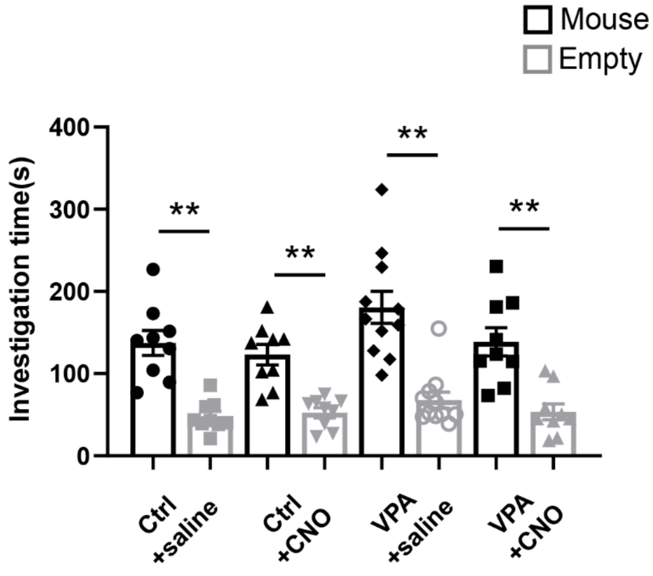

Supplementary figure 2

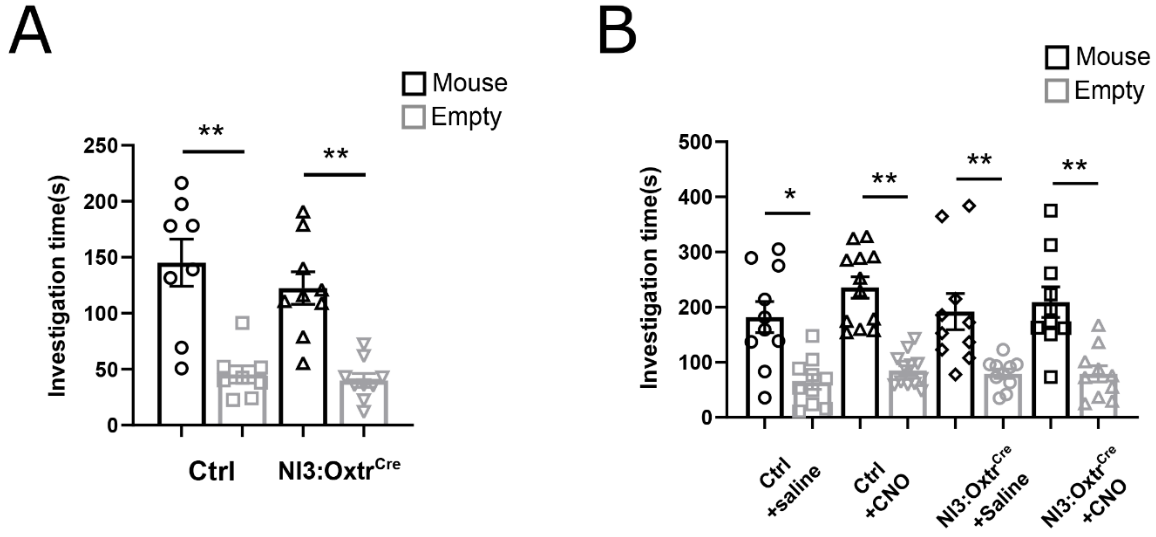

Supplementary figure 3

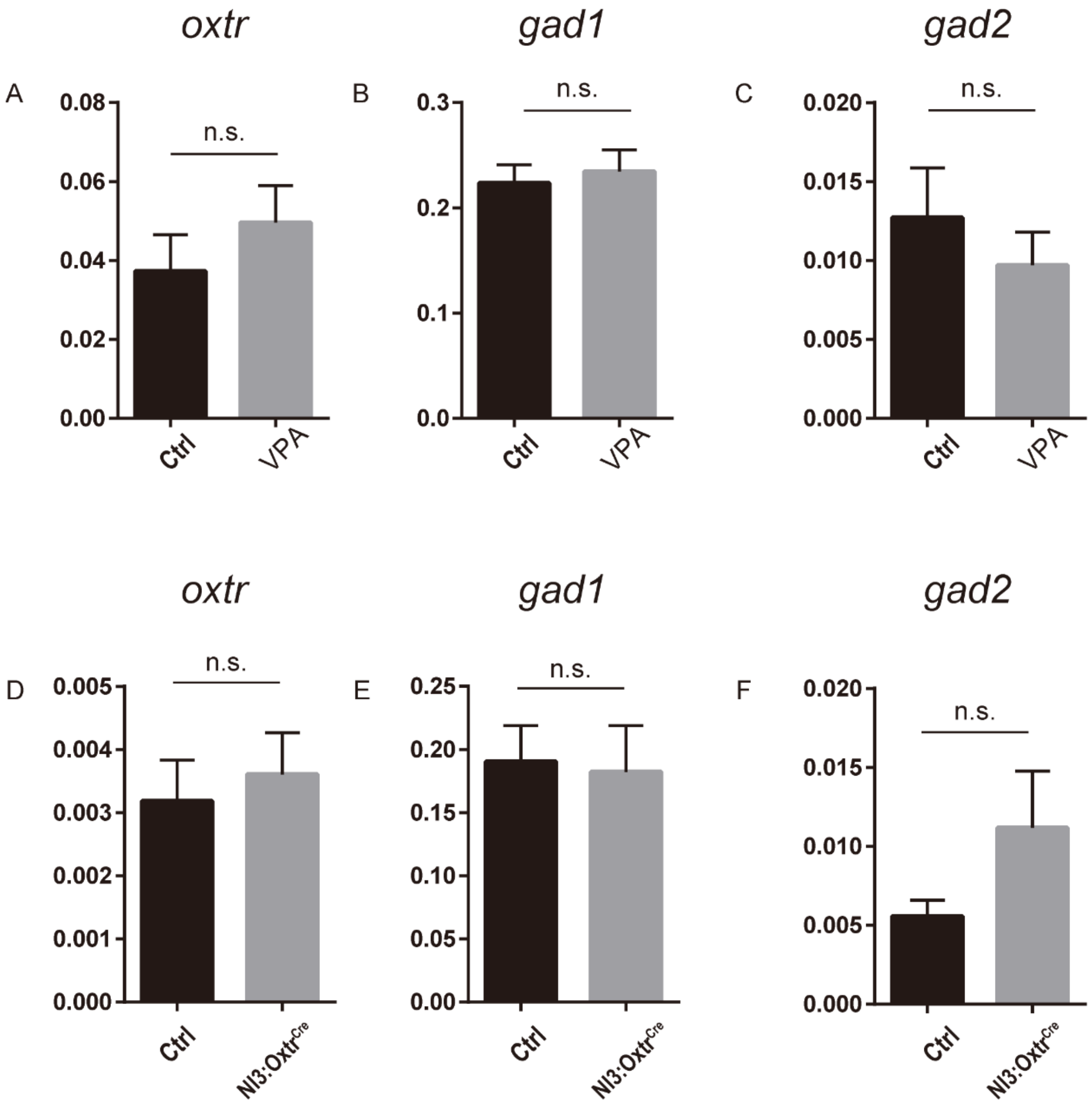

Supplement: Supplementary file 1 — Supplementary Figures. [file 41598_2020_79109_MOESM1_ESM.pdf]
